# A Neural-Dynamic Architecture for Concurrent Estimation of Object Pose and Identity

**DOI:** 10.3389/fnbot.2017.00023

**Published:** 2017-04-28

**Authors:** Oliver Lomp, Christian Faubel, Gregor Schöner

**Affiliations:** ^1^Institut für Neuroinformatik, Ruhr-University Bochum, Bochum, Germany

**Keywords:** object recognition, pose estimation, neural dynamics, recurrent process, top-down feedback

## Abstract

Handling objects or interacting with a human user about objects on a shared tabletop requires that objects be identified after learning from a small number of views and that object pose be estimated. We present a neurally inspired architecture that learns object instances by storing features extracted from a single view of each object. Input features are color and edge histograms from a localized area that is updated during processing. The system finds the best-matching view for the object in a novel input image while concurrently estimating the object’s pose, aligning the learned view with current input. The system is based on neural dynamics, computationally operating in real time, and can handle dynamic scenes directly off live video input. In a scenario with 30 everyday objects, the system achieves recognition rates of 87.2% from a single training view for each object, while also estimating pose quite precisely. We further demonstrate that the system can track moving objects, and that it can segment the visual array, selecting and recognizing one object while suppressing input from another known object in the immediate vicinity. Evaluation on the COIL-100 dataset, in which objects are depicted from different viewing angles, revealed recognition rates of 91.1% on the first 30 objects, each learned from four training views.

## 1 Introduction

Imagine you are sitting at a table on which a number of objects are distributed. Perhaps you are trying to repair a toaster and have tools, parts, and the toaster spread out in front of you. You will easily commit to memory the parts that you remove upon disassembly of the unit, even if they are objects that you have never seen before in your life. Your repair will be successful only if you recognize these objects even if they now lie in a different pose before you. You will be able to grasp and handle them precisely because you are able to estimate their pose. When objects move, perhaps through your own action, you will be able to update the pose estimate and the associated object representations in your memory, grasping the shifted or rotated object correctly.

In the lab, psychophysical studies have shown that in the context of a natural scene, human observers are able to commit large numbers of objects to memory by examining each object only once for a short time (Hollingworth, 2005). In the recall phase, participants were asked to determine an object’s identity by discriminating it against other instances of the same category and to estimate the object’s pose by discriminating it against other rotated poses of the same instance. Participants had very high retention rates even when tests were made a day later. Representations of object pose can be continuously linked to sensory inputs. Fast online updating of object-oriented movements to translations or rotations of an object, for instance, have been documented even under conditions in which the change of object pose was not consciously perceived, because the change was induced during a visual saccade when observers are blind to visual input (Prablanc and Martin, 1992).

The capacity to recognize individual object instances and estimate object poses contrasts with the human perceptual skill of detecting objects of a given category in a visual scene. Humans can do this with exposure times as short as 20 ms and response times as short as 150 ms (Thorpe et al., 1996), suggesting that this is done primarily through feedforward neural processing. Such fast visual categorization works for superordinate categories and does not necessarily come with information about the specific feature values or the location of the object (Mack and Palmeri, 2011).

Because of the good performance of the primate visual system, it often serves as an inspiration for artificial vision systems. Most neurally inspired work on object recognition has taken the feedforward picture (Fukushima, 1980; LeCun and Bengio, 1995; LeCun et al., 1998; Riesenhuber and Poggio, 1999; Wiskott and Sejnowski, 2002; Serre et al., 2007). The focus of such work has been the discriminative power of the high-dimensional feature vectors thought to be represented in primate visual cortex (Serre et al., 2007). The organization of visual processing in cortex into a hierarchy of processing steps has inspired hierarchical approaches in artificial vision that forms the basis for invariance, the classification of visual inputs as pertaining to the same class as the visual object varies in surface appearance through differences in lighting or pose. The HMAX model (Riesenhuber and Poggio, 1999; Serre et al., 2007), for instance, pools the outputs of units that model orientation-tuned simple cells in visual cortex, passing only the maximal activation along to complex cells on different scales. In this model, the units in the final layer of the hierarchy are thus invariant to pose. Feedforward approaches do not necessarily discard pose information, though. In slow feature analysis (Wiskott and Sejnowski, 2002), a hierarchy compresses the high-dimensional image information into the most temporally invariant components. Some of these components code for the object’s identity, while others encode information about the object’s pose.

In terms of recognition performance, deep neural networks (DNNs), first introduced by Fukushima (1980) and extended by LeCun and Bengio (1995), LeCun et al. (1998), and Hinton et al. (2006), have recently achieved very high classification rates that approach and even exceed human abilities (see, for example, Ciresan et al. (2012)). Pose information is typically discarded in these networks, although it is possible, in principle, to address pose (Osadchy et al. (2007); albeit in a face detection rather than recognition task). DNNs assign an object to classes that are learned from many example instances. Probabilistic approaches to object categorization have reduced the number of training examples required for successful categorization (Fei-Fei et al., 2003). However, when classifying, multiple instances will always be required to learn the properties defining the classes. Correspondence-based approaches, in contrast, can sometimes work from individual instances. They establish correspondence by matching landmarks on a stored and a current image (Wiskott et al., 1997; Zhu and von der Malsburg, 2004). Establishing correspondence is, in general, difficult and computationally costly. It works best when landmarks are highly characteristic, as is the case for face recognition, where correspondence-based approaches have been very successful (Wiskott et al., 1997). A correspondence-based approach that employs the loosely neurally inspired SIFT features comes close to what we aim to achieve here in that it explicitly estimates pose during recognition (Lowe, 2004). Correspondence is established for keypoints using nearest neighbor methods based on the SIFT descriptors. Integration across keypoints makes use of a generalized Hough transformation that identifies clusters of features that vote for the same pose. The pose emerges from an optimal inverse of an over-determined system of linear transformation equations.

Cognitive robots, especially when they interact with humans, must have the capacities we outlined for humans in the “toaster scenario” above: recognizing object identity from a single previous view, estimating an object’s pose relative to that view, and updating that estimate as the object is moved (Kragic et al., 2005). These capacities are critical to object manipulation, in particular, in scenarios in which a robot interacts with a human user who may be unwilling to provide a lot of training data to the robot, even while expecting the robot to discriminate between object identities that are not captured by well-known object categories (e.g., different screw drivers on the work surface). Moreover, human interaction typically involves users handling objects, leading to dynamic scenes wherein pose information may change even while processes for pose estimation and object recognition are active.

While object recognition in robotic scenarios is a focus of current work (e.g., Schoeler et al. (2014) and Pasquale et al. (2015)), the problem of combining object recognition with pose estimation in such scenarios has not yet been sufficiently addressed. This is the focus of our contribution. We exploit analogies with neural mechanisms of vision based on three ideas. First, in our system, we represent objects as localized histograms of color and of edge orientations, as well as heuristic measures of object shape. Our system learns these features for a single instance of an object by storing and associating them with an object label. We choose this representation to enable the active transformation of an object’s stored image into new poses observed in novel images. Second, inspired by the map-seeking circuit (Arathorn, 2002; Gedeon and Arathorn, 2007), we solve the two problems of identifying objects and estimating their pose in the image plane simultaneously in a recurrent loop that bootstraps initial, broad estimates to the final values over time (for a related attempt to use the map-seeking circuit in neurally inspired vision, see Wolfrum et al. (2008), who use it to recognize faces in different poses). Third, we realize this bootstrapping process via attractors of a neural dynamics. This enables the continuous online updating of the pose estimate in response to object movement and stabilizes object identity in response to time-varying or fluctuating inputs.

By combining a “what” with a “where” channel of visual processing (Milner and Goodale, 1995) in a closed loop, this model moves beyond the feedforward neural networks that are the basis for most neurally inspired solutions to problems in artificial vision. To achieve this, we employ principles of neural dynamics, neural networks in which recurrent connections dominate, as formalized in Dynamic Field Theory (DFT; Schöner et al. (2015)). A key idea is that both recognition and estimation are selection decisions that are realized within the recurrent neural dynamics that combine the two streams. Adhering strictly to the neural principles of DFT and eschewing algorithmic components or “read-out” procedures, the proposed model is one large (neural) dynamical system. In assessing its performance in settings close to those of technical computer vision, we demonstrate that the visual function of object recognition with pose estimation can be obtained from the postulated neural principles.

Preliminary results were published in Faubel and Schöner (2008, 2009, 2010) and Faubel (2009). The model is available online along with a manual.^1^

## 2 Materials and Methods

**Figure 1:**
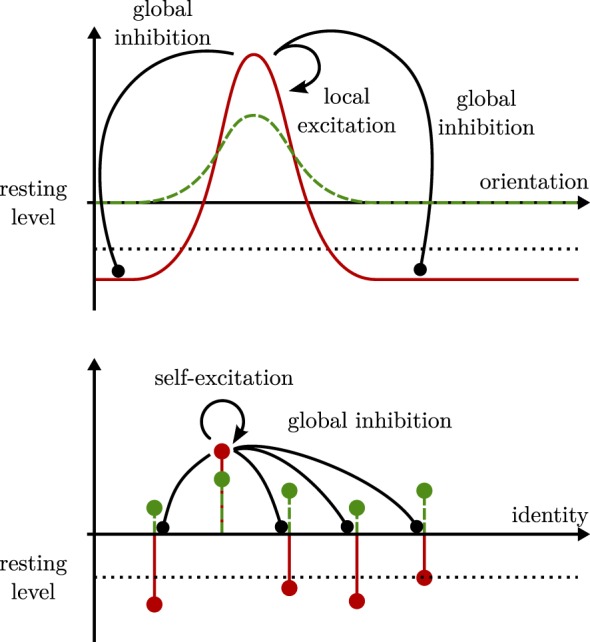
**Dynamic neural fields (top panel) form localized regions of suprathreshold activation (solid red line) in response to localized input (dashed green line)**. The shape of these peaks depends on local excitation (solid black arrow) and global inhibition (solid black line ending in a circle). Dynamic neural nodes (bottom panel) follow similar dynamics. Their activation and input, here, are pictured along a categorical identity axis and symbolized by circles with stems.

Pose is represented neurally through dynamic neural fields, such as illustrated in Figure 1 (top panel). Neural fields capture population distributions of activation as they arise in cortex and other brain structures (Lins and Schöner, 2014). They are modeled as activation distributions over relevant metric dimensions, here, the two-dimensional position of an object or the orientation of an object relative to a reference image. Localized peaks of activation are stable solutions of a neural dynamics of these activation fields, introduced below. The locations over which such peaks are generated encode the estimate of the underlying dimension. The peaks are stabilized by neural interaction within the neural fields, locally excitatory and globally inhibitory. Such connectivity also characterizes the competitive dynamics of neural nodes that are used to represent object identity (Figure 1, bottom panel).

**Figure 2:**
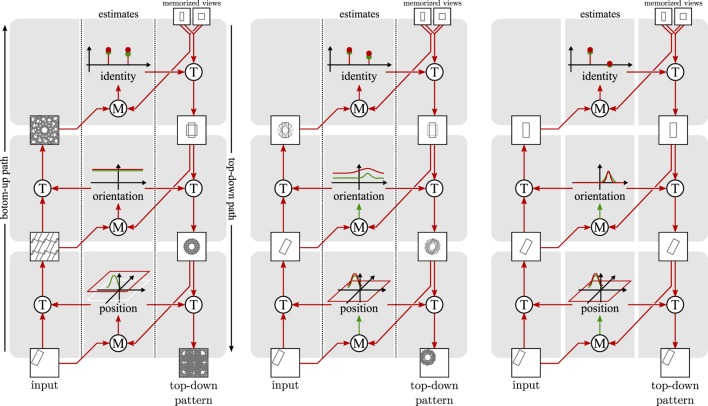
**Three snapshots (from left to right) of the architecture during the convergence process**. Circles with a “T” stand for transformation operations, while circles with an “M” stand for matching operations. The estimates use neural fields depicted as in Figure 1. The position estimate extends this graphical representation to two dimensions. The left panel shows an initial state where pose and identity are unspecific. The middle panel shows an intermediate state where the position estimate has become specific, but identity and orientation estimates are still largely unspecific. In the right panel, the architecture has converged. Both identity and pose estimates are specific.

These two types of neural dynamics for object pose and identity form the core of the neural-dynamic architecture illustrated in Figure 2. A bottom-up path transforms an input image based on the current estimate of position and, in a next stage, of orientation. These transformations align the pose of the object in the input image with learned object views, enabling matching for recognition. A top-down path matches the recognized image with every possible rotation of the input to obtain an estimate of orientation. In a next stage, the top-down path reverses the estimated rotation of the learned view and matches it with every possible translation of the input to obtain an estimation of position.

Figure 2 illustrates how the recurrent loop of bottom-up and top-down neural processing converges toward estimates of object pose and identity. On the left, an early stage of convergence is shown, in which the neural fields representing pose have not yet converged to localized peaks of activation. The transformations in the bottom-up path then generate a superposition of all possible translations and rotations of the input image. The neural nodes representing object identity have not converged toward a winner either, so that the top-down path generates superposed views of all learned object views. In the middle panel, the neural field representing object position has converged to a localized peak, so that along the bottom-up path a localized image patch is generated. The neural field representing orientation is just beginning to converge, so that the transformed image is still rotationally blurred to some extent. The neural nodes representing object identity are also beginning to converge, so that the learned view along the top-down path begins to resemble an individual object, improving the precision of the matches of object pose. In the right panel, the recurrent loop has converged, with a sharp neural activation peak for object position and orientation and a winner-takes-all representation of object identity. The bottom-up path shifts and rotates the input image to center the object in the upright pose in which it was learned. The top-down path reverses these transformations and correctly predicts the input image.

**Figure 3:**
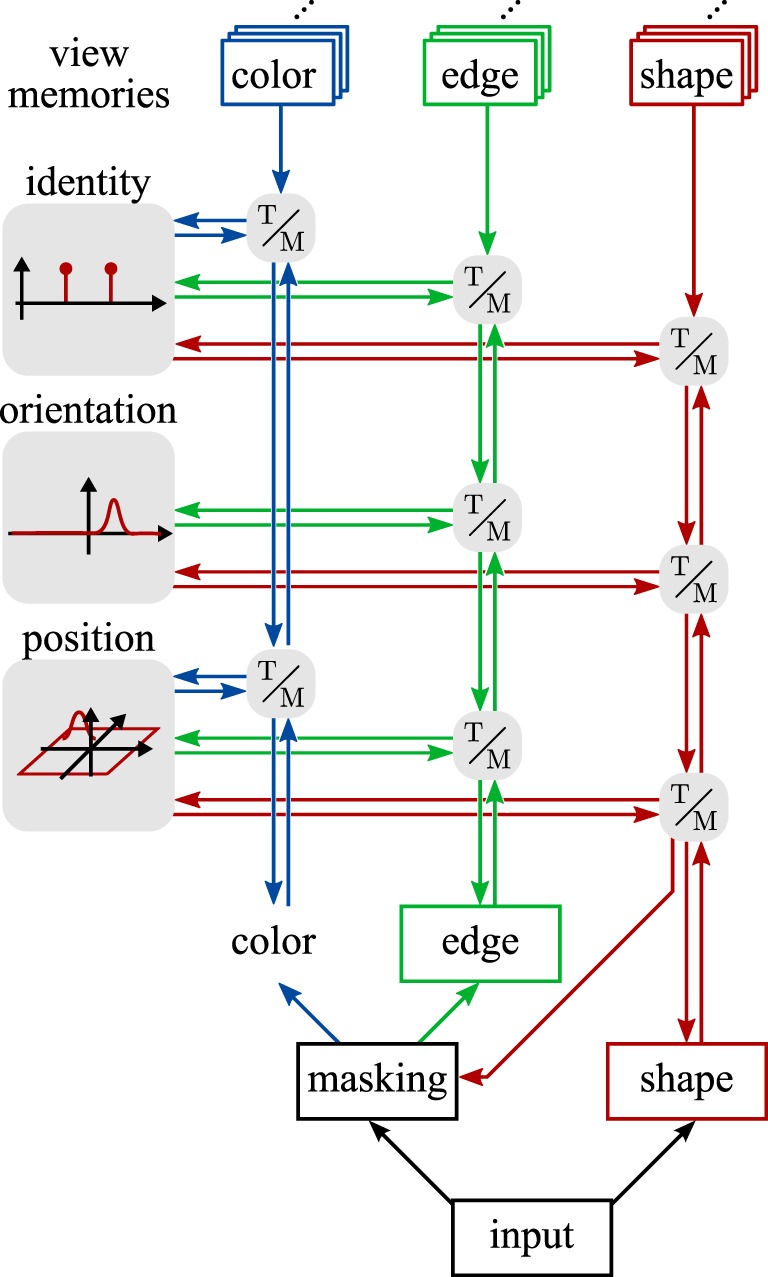
**An overview of the contributions of the feature channels to the estimation process**. The edge channel here is representative of three channels of edge orientations extracted from the luma (Y), blue (Cb), and red (Cr) chromaticity channels of the YCbCr color model. Gray boxes labeled “T/M” stand for transformation and matching stages as elaborated in Figure 2 and the text.

Figure 2 only illustrates one of five feature channels, which the full model uses in parallel. The shape channel illustrated in the figure plays an important role for segmentation but has limited discriminative power because we use a coarse shape representation. We explain the neural dynamics of the bottom-up and top-down paths for the shape channel in exemplary fashion next. The neural dynamics of the other feature channels are analogous and are summarized in a separate subsection. This is followed by an account for how the different feature channels are fused around Figure 3. We close the materials and methods section with notes about the process of learning objects and a description of the performance measures used to evaluate the model.

### 2.1 The Shape Channel

**Figure 4:**
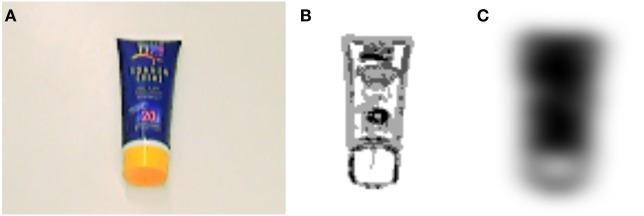
**Example for the shape extracted from an input image**. Panel **(A)** shows the input image and **(B)** the sum of the energies of edges in the black (Y) and chromaticity (Cr and Cb) channels of the input image. Panel **(C)** shows The final extracted shape. In the latter two figures, darker areas are more active than lighter areas.

The shape channel is based on responses from a bank of oriented, steerable edge filters (Freeman and Adelson, 1991). First, we extract the edge energy separately for the black (Y) and chromaticity (red, Cr and blue, Cb) channels. We then sum these edge energies, and partially fill out the result by applying Gaussian smoothing. This is followed by thresholding and range compressions with logarithmic functions to finally give a shape image *I*(*x,y,t*) ↦ ℝ for a given input image at time *t* ∈ ℝ^+^ (see Figure 4).

We first explain the bottom-up, then the top-down path. Next, the neural dynamics at the core of the closed loop is explained for pose and then for identity estimation. To the purpose of this exposition, we assume that the system has already been trained and that all training views are in a canonical pose.

#### 2.1.1 The Bottom-up Path: Matching Object Views

We estimate the parameters of a set of transformations, *T* = {*sh* = shift, *rot* = rotation}, that describe translation (shift) and rotation of the image. Each pose parameter is represented by an activation function, *p_s_*: *R_s_* × ℝ^+^ ↦ [0, 1], defined over the range of possible pose parameter values, *R_s_* ⊆ ℝ*^d^* of transformation *s* ∈ *T*, and time (the function for *p_s_* is given in equation (15)). The higher the activation level of a pose parameter value, the better the match achieved by transforming the image view to the corresponding pose.

The input pattern is first transformed according to the current shift estimate *p*_sh_:

(1) $$I_{\text{bu}}^{\text{sh}}\left(x,y,t\right)=\iint p_{\text{sh}}\left(x-x^{\prime },y-y^{\prime },t\right)I\left(x^{\prime },y^{\prime },t\right)\;{dx}^{\prime }\;{dy}^{\prime }.$$

Mathematically, this is analogous to the convolution of the function *I* with the kernel *p*_sh_. The convolution can be visualized as a superposition of all possible transformed versions of the input, *I*(*x*′, *y*′, *t*), each weighted by the shift representation, *p*_sh_(*x* − *x*′, *y* − *y*′, *t*).

**Figure 5:**
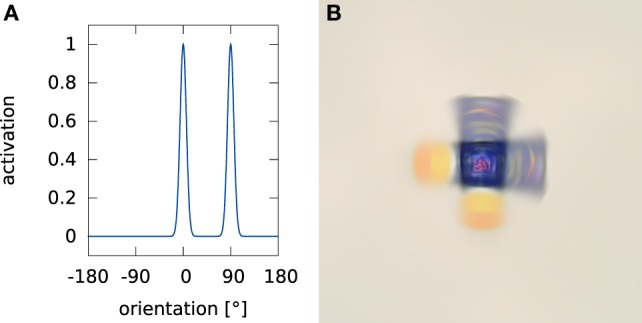
**An example of a weighted superposition**. Panel **(A)** shows an example rotation estimate where two poses (0° and 90°) are equally active. Panel **(B)** shows the result of transforming an exemplary input image by this rotation estimate.

After applying the shift estimate, we change the coordinate system for $I_{\text{bu}}^{\text{sh}}$ to log-polar coordinates (which we denote by ρ for the distance from the center and by ϕ for the angle). This allows us to rotate the input image by transforming along these coordinate axes. Thus,

(2) $$I_{\text{bu}}^{\text{rot}}\left(\rho ,\phi ,t\right)=\int p_{\text{rot}}\left(\phi -\phi^{\prime },t\right)I_{\text{bu}}^{\text{sh}}\left(\rho ,\phi^{\prime },t\right)\;{d\phi }^{\prime },$$

is the rotated version of the shifted pattern. Again, we use the idea of a convolution as a weighted superposition. Figure 5 shows an example of such a superposition when the current orientation estimate is bimodal because it has not yet converged on a unique estimate. By going back to Cartesian coordinates for $I_{\text{bu}}^{\text{rot}}$, we obtain the shifted and rotated input image.

We next compare the transformed input image, $I_{\text{bu}}^{\text{rot}}$, with all learned object views, *W_l_*(*x, y, t*), where *l* = 1, 2, … corresponds to the labels given to the learned object views. For each known object view, this provides a match value

(3) $${\text{match}}_{l}\left(t\right)=\iint {\hat{I}}_{\text{bu}}^{\text{rot}}\left(x,y,t\right){\;\hat{W}}_{l}\left(x,y,t\right)\;\mathrm{dx}\;\mathrm{dy}.$$

Here, we use the notational convention that

(4) $$\hat{X}\left(\text{x},t\right)=\frac{X\left(\text{x},t\right)-\bar{X}}{∥X{∥}_{2}}$$

where $\bar{X}$ is the mean value (disregarding time) of *X*: ℝ*^n^* × ℝ^+^ ↦ ℝ (for *n* ≥ 1) and ||*X*||_2_ is the L2 norm of *X* (also disregarding time). Thus, ${\hat{I}}_{\text{bu}}^{\text{rot}}$ is the mean-free, normalized version of $I_{\text{bu}}^{\text{rot}}$. The match values, match*_l_*(*t*), serve as input to a set of dynamic neural nodes which detect and select candidates from the learned views as described in Section 2.2.1.

#### 2.1.2 The Top-down Path: Matching Pose

In the top-down path, the learned views, *W_l_*, are superposed,

(5) $$P_{\text{td}}\left(x,y,t\right)=\sum_{l\in L}\;p_{l}\left(t\right)W_{l}\left(x,y,t\right),$$

weighted by their match values *p_l_*(*t*) ∈ [0, 1] (specified in detail in equation (20)). The result is matched against the transformed versions of the input image in the bottom-up path, yielding new pose estimates. We first match the superposition (in log-polar coordinates) with the shifted input image along the angle parameter:

(6) $${\text{match}}_{\text{rot}}\left(\phi ,t\right)=\iint {\hat{P}}_{\text{td}}\left(\rho^{\prime },\phi +\phi^{\prime },t\right){\hat{I}}_{\text{bu}}^{\text{sh}}\left(\rho^{\prime },\phi^{\prime },t\right)\;{d\rho }^{\prime }\;{d\phi }^{\prime }.$$

This match value serves as input to the dynamic neural field that represents the rotation of the object (see Section 2.2.1).

To compute the spatial match, we first transform *P*_td_ back to the original orientation based on the rotation representation, *p*_rot_, now used as an inverse:

(7) $$P_{\text{td}}^{\text{rot}}\left(\rho ,\phi ,t\right)=\int p_{\text{rot}}\left(\phi^{\prime }-\phi ,t\right)P_{\text{td}}\left(\rho ,\phi^{\prime },t\right)\;{d\phi }^{\prime }.$$

Because we formulated rotation transformation as a simple shift operation, we find its inverse by reversing the argument, $p_{\text{rot}}^{\text{inv}}\left(\phi -\phi^{\prime },t\right)=p_{\text{rot}}\left(\phi^{\prime }-\phi ,t\right)$. The rotated image is correlated with the input pattern

(8) $${\text{match}}_{\text{sh}}\left(x,y,t\right)=\iint {\hat{P}}_{\text{td}}^{\text{rot}}\left(x+x^{\prime },y+y^{\prime },t\right){\hat{I}}_{\text{bu}}\left(x^{\prime },y^{\prime },t\right)\;{dx}^{\prime }\;{dy}^{\prime }.$$

This match value serves as input to the object position field (see Section 2.2.1).

### 2.2 Neural Dynamics

The cores of the model are neural-dynamic representations of pose and of object identity, that implement processes of pose estimation and object recognition. We review these in turn.

#### 2.2.1 Dynamic Neural Fields for Pose Representation

Representing pose neurally makes it possible to perform pose transformations before a specific pose estimate has been obtained. Initially, the neural field representation of pose is flat, so that all possible values of the pose parameter are equally valid. This representation evolves in time as described by dynamic neural fields, ultimately converging to mono-modal distributions, whose peak locations indicate the final pose estimate. This convergence occurs at the same time as the estimate of object identity also evolves and converges, as sketched below.

We explain the neural representation of pose, described by the pose parameter *r*, jointly for the two transformations translation and rotation. For translation, **r** = (*x, y*) ⊆ ℝ^2^; and, for rotation, *r* = ϕ ∈ [0, π).

To represent the value of each pose parameter, we introduce two layers of dynamic neural fields (DNFs), *u*_1_(*r, t*) and *u*_2_(*r, t*). (Strictly speaking, all fields and associated parameters need to have an index to distinguish the two transformations. To avoid clutter, we suppress these indices.) The activation in both layers is governed by a neural dynamics inspired by classical work of Amari (1977). For the first layer, the dynamics is given by

(9) $$\tau_{1}{\dot{u}}_{1}\left(r,t\right)=-u_{1}\left(r,t\right)+h_{1}+s_{1}\left(r,t\right)+c_{\eta ,1}\;\eta_{1}\left(r,t\right)\;+\int w_{1}\left(r-r^{\prime }\right)\sigma \left(u_{1}\left(r^{\prime },t\right)\right)\;{dr}^{\prime },$$

where *h*_1_ < 0 is the resting level to which activation relaxes in the absence of any input, *s*_1_, on the timescale τ_1_ > 0. η_1_ is a Gaussian white noise term with unit variance, weighted by the factor *c_η_*_,1_ ≥ 0. Only field sites with significant activation levels contribute to interaction, as described by a sigmoid function *σ*: ℝ ↦ [0, 1],

(10) $$\sigma \left(u\right)=\frac{1}{2}\left(1+\frac{\beta u}{1+\beta |u|}\right),$$

with steepness β > 0. The sign and size of interaction is defined by an interaction kernel,

(11) $$w_{i}\left(r-r^{\prime }\right)=\sum_{j}\;a_{j}g_{\sigma_{j}}\left(r-r^{\prime }\right)-\gamma_{i}$$

which consists of global inhibition, γ*_i_* ≥ 0, and local interaction of strength *a_j_* (>0 for local excitation, <0 for local inhibition), modeled as a sum of Gaussians

(12) $$g_{\sigma_{j}}\left(r\right)=\text{exp}\left[-\frac{r^{2}}{2\sigma_{j}^{2}}\right]\;,$$

with width σ*_j_*. The match functions defined in Section 2.1.2 provide input, *s*_1_, into the corresponding first layer of neural activation: for translation, *s*_1_(*x, y*) = match_sh_(*x, y*), and for orientation, *s*_1_(ϕ) = match_rot_(ϕ).

This variant of neural dynamics was analyzed by Amari (1977) who showed that for sufficiently strong localized inputs, localized connected regions of suprathreshold activation (*peaks*) become attractor states. The shape of these peaks is largely determined by the interaction kernel. Local excitation (positive *a_j_* in equation (11)) strengthens peaks beyond the local level of input. Peaks suppress all other field sites through global inhibition (γ*_i_* > 0 in equation (11)), which can lead to selection in which only a single peak may form within a field.

The dynamics of the second layer is given by

(13) $$\begin{array}{ll} \tau_{2}{\dot{u}}_{2}\left(r,t\right) & =-u_{2}\left(r,t\right)+h_{2}+c_{\eta ,2}\;\eta_{2}\left(r,t\right)\\ & \;\;+\int w_{2}\left(r-r^{\prime }\right)\sigma \left(u_{2}\left(r^{\prime },t\right)\right)\;{dr}^{\prime }\\ & \;\;+c_{12}\int g_{\sigma_{12}}\left(r-r^{\prime \prime }\right)\theta \left(u_{1}\left(r^{\prime \prime },t\right)\right){dr}^{\prime \prime }. \end{array}$$

It receives input from the first layer controlled by the coupling strength, *c*_12_ > 0, and the semi-linear threshold function,

(14) $$\begin{array}{l} \theta \left(u\right)=\left\{\begin{array}{ll} u:\; & u\geq 0\\ 0:\; & \text{otherwise} \end{array}\right. \end{array}$$

All other variables are defined analogously to equation (9).

The actual pose estimate represented by the two-layer structure is a multiplicative mixture of the output of the two layers,

(15) $$p_{s}\left(r,t\right)=m\left(t\right)\sigma \left(u_{2}\left(r,t\right)\right)+\left(1-m\left(t\right)\right)\theta \left(u_{1}\left(r,t\right)\right),$$

where *s* is an index for a transformation. Here, *m*(*t*) = σ(*u_p_*(*t*)) is the ratio of the mixture. Its value depends on the activation of a peak detector, *u_p_*(*t*), which is governed by the dynamics

(16) $$\tau_{p}{\dot{u}}_{p}\left(t\right)=-u_{p}\left(t\right)+w_{p}\sigma \left(u_{p}\left(t\right)\right)+\int \sigma \left(u_{2}\left(r^{\prime },t\right)\right){dr}^{\prime }+h_{p},$$

where *w_p_* > 0 and *h_p_* < 0.

In the first layer, only weak global inhibition is applied so that multiple candidate values of pose may become active in the field. Strong candidates are strengthened by local excitation, while very weak candidates are suppressed through global inhibition. Global inhibition is stronger in the second layer, so that it selects one of the candidate pose values from the first layer as the final pose estimate.

We choose τ_1_ < τ_2_, so that the first layer converges faster than the second layer. The fields will thus initially represent a set of candidate pose parameter values. These candidates are used to transform the input image and the superposition of the learned views leading to increasingly accurate pose estimates. This process continues until the second layers decide which candidate values to use for the final pose estimate.

#### 2.2.2 Dynamic Neural Nodes for Identity Representation

Each learned object view is indexed by a label *l*. We represent the estimation of the best-matching object view by the activation of two layers of dynamic neural nodes, *u_l_*_,1_ and *u_l_*_,2_. The dynamics for these layers of nodes are defined analogously to the dynamics of the neural activation fields used for pose representation. For the first layer, the dynamics are given by

(17) $$\begin{array}{ll} \tau_{1}{\dot{u}}_{l,1}\left(t\right) & =-u_{l,1}\left(t\right)+h_{1}+{\text{match}}_{l}\left(t\right)+c_{\eta ,1}^{\text{L}}\eta_{1}\left(l,t\right)\\ & \;\;+\sum_{l^{\prime }}\;w_{1,l,l^{\prime }}\sigma \left(u_{l^{\prime },1}\left(t\right)\right), \end{array}$$

where τ_1_ > 0 is the time scale, *h*_1_ ≤ 0 is the resting level, η_1_ is a Gaussian white noise term with unit variance, $c_{\eta ,1}^{\text{L}}$ ≥ 0, and

(18) $$\begin{array}{l} w_{i,l,l^{\prime }}=\left\{\begin{array}{ll} w_{i,\text{self}}:\; & l=l^{\prime }\\ \gamma_{i}:\; & \text{otherwise} \end{array}\right. \end{array}$$

is the interaction matrix with self-excitation strength *w_i_*_,self_ > 0 and global inhibition γ*_i_* ≤ 0.

The second layer follows the dynamics

(19) $$\begin{array}{ll} \tau_{2}{\dot{u}}_{l,2}\left(t\right) & =-u_{l,2}\left(t\right)+h_{2}+\sum_{l^{\prime }}\;w_{2,l,l^{\prime }}\sigma \left(u_{l^{\prime },2}\left(t\right)\right)+c_{\eta ,2}^{\text{L}}\eta_{2}\left(l,t\right)\\ & \;\;+c_{12}\theta \left(u_{l,1}\left(t\right)\right), \end{array}$$

where τ_2_ > 0 is the time scale, *h*_2_ ≤ 0 is the resting level, η_2_ is a Gaussian white noise term with unit variance, $c_{\eta ,2}^{\text{L}}$ ≥ 0, the weights, *w*_2,*l,l*′_, are defined as in equation (18), and *c*_12_ > 0 is the connection strength from layer one to layer two.

The actual estimate of object identity represented by the two-layer structure is a multiplicative mixture of the output of the two layers,

(20) $$p_{l}\left(t\right)=m\left(t\right)\sigma \left(u_{l,2}\left(r,t\right)\right)+\left(1-m\left(t\right)\right)\theta \left(u_{l,1}\left(r,t\right)\right).$$

As before, *m*(*t*) = σ(*u_p_*(*t*)) is the ratio of the mixture. Its value depends on the activation of a detector for active label nodes, *u_p_*(*t*), which is governed by the dynamics

(21) $$\tau_{p}{\dot{u}}_{p}\left(t\right)=-u_{p}\left(t\right)+w_{p}\sigma \left(u_{p}\left(t\right)\right)+\sum_{l^{\prime }}\;\sigma \left(u_{l^{\prime },2}\left(t\right)\right)+h_{p},$$

where *w_p_* > 0 and *h_p_* < 0.

In analogy to the two-layer structure of the pose representation, the first layer has only little global inhibition and detects candidates for the best-matching views. The second layer is much slower than the first one (τ_1_ < τ_2_), and is much more selective (γ_1_ < γ_2_), allowing only a single view node to become active. The final recognition, then, is given by the active node l* for which σ (*u*_l*,2_(*t*)) >> 0.

### 2.3 The Color and Edge Channels

Shape is combined with feature channels for color (hue of the HSV color model) and for the orientation of edges of the luma (Y) and chromaticity (Cb, Cr) components of the image (Figure 3). These feature channels are based on localized histograms that are explained next. We then describe how the pose transformations for translation and for rotation are performed on these histograms, followed by a description of how the transformed histograms are matched to the top-down path.

#### 2.3.1 Histogram Extraction

For computational efficiency, localized histograms for each feature channel, *F*, are extracted only on a regular two-dimensional grid of image points that subsample the image. These grid points become the centers, **c***_i,j_* ∈ ℝ^2^ of the histograms, which are thus labeled by two discrete indices, *i*, and *j*:

(22) $$h_{i,j}\left(f,t\right)=n_{F}\left(t\right)\iint g_{\sigma_{h},{\text{c}}_{i,j}}\left(x,y\right)\;m_{F}\left(x,y,t\right)\times \chi_{\left[f,f+\Delta f\right]}\left(I_{F}\left(x,y,t\right)\right)\;\mathrm{dx}\;\mathrm{dy}$$

Here, *I_F_* : ℝ^2^ × ℝ^+^ ↦ ℝ are the feature values extracted from the input image *I*. χ_[*f,f*+Δ*f*]_(⋅) is the characteristic function of an interval, [*f*, *f* + Δ*f*], along the feature dimension. It returns 1 when the argument falls into the interval and zero else. The size of this interval, Δ*f*, reflects the discrete sampling of the feature dimension used in practice. Here, we still keep the continuous notation for the feature dimension, *f*, because that makes it easier to express the pose transformations in feature space. $g_{\sigma_{h},{\text{c}}_{i,j}}\left(x,y\right)$ is a Gaussian kernel

(23) $$g_{\sigma_{h},{\text{c}}_{i,j}}\left(x,y\right)=\text{exp}\left[-\frac{∥\left(x,y\right){-{\text{c}}_{i,j}∥}_{2}^{2}}{2\sigma_{h}^{2}}\right],$$

centered on the grid points, **c***_i,j_*. The masking term $m_{F}\left(x,y,t\right)=m_{F}^{\theta }\left(x,y,t\right)P_{\text{td}}^{\text{sh}}\left(x,y,t\right)$, where

(24) $$P_{\text{td}}^{\text{sh}}\left(x,y,t\right)=\iint p_{\text{sh}}\left(x^{\prime }-x,y^{\prime }-y,t\right)P_{\text{td}}^{\text{rot}}\left(x^{\prime },y^{\prime },t\right)\;{dx}^{\prime }\;{dy}^{\prime }$$

is the shape predicted in the top-down path ($P_{\text{td}}^{\text{rot}}$, see equation (7)), and $m_{F}^{\theta }\left(x,y,t\right)\in \left\{0,1\right\}$ is a threshold function. For color, this threshold is

(25) $$\begin{array}{l} m_{\text{col}}^{\theta }\left(x,y,t\right)=\left\{\begin{array}{ll} 1:\; & \text{saturation}\left(x,y,t\right)>\theta_{\text{sat}}\\ \; & \wedge \;\text{value}\left(x,y,t\right)>\theta_{\text{val}}\\ 0:\; & \text{otherwise} \end{array}\right., \end{array}$$

where saturation (*x, y, t*) and value (*x, y, t*) are given by the HSV color model, and θ_sat_ ∈ ℝ and θ_val_ ∈ ℝ are thresholds. Analogously, the threshold for the edge channels is defined by

(26) $$\begin{array}{l} m_{\text{edge}}^{\theta }\left(x,y,t\right)=\left\{\begin{array}{ll} 1:\; & \text{energy}\left(x,y,t\right)>\theta_{\text{edge}}\\ 0:\; & \text{otherwise} \end{array}\right., \end{array}$$

where energy (*x, y, t*) is the edge energy obtained from steerable filters (Freeman and Adelson, 1991) and θ_edge_ ∈ ℝ is a threshold. Finally, the normalization factor, *n_F_*, is given by

(27) $$n_{F}\left(t\right)={\left(\iint m_{F}^{\theta }\left(x,y,t\right)\;\mathrm{dx}\;\mathrm{dy}\right)}^{-1}.$$

#### 2.3.2 Translation

The goal is to match the learned object views with those parts of the image that the current shift representation, *p*_sh_, maps onto the center of the visual array. In a sense, the learned views can be thought of as being localized at that center. The shift representation is thus used to translate image patches to the center and at the same time as a mechanism of spatial attention that selects the portions of the image to be matched. The spatial range over which image information is taken into account is determined by the spatial distribution of activation in the shift representation itself.

Implementing this idea is complicated by the fact that we subsampled the image on a coarse subgrid for efficiency reasons (see above). We must, therefore, subsample the shift representation, *p*_sh_, in the same way. To do this without loss of local peaks, we use a maximum filter. Specifically, $p_{\text{sh}}^{\text{max}}\left({\text{c}}_{i,j},t\right)$, is determined by finding the maximum activation level of the shift representation, *p*_sh_, within a square sample centered on the grid point, **c***_i,j_*. Attentional selection together with shift transformation is then achieved through a weighted sum of the localized histograms:

(28) $$h_{\text{bu}}^{\text{sh}}\left(f,t\right)=\sum_{i,j}\;p_{\text{sh}}^{\text{max}}\left({\text{c}}_{i,j},t\right)h_{i,j}\left(f,t\right).$$

This yields a single “bottom-up” histogram of the feature dimension, *F*, that the variable, *f*, samples.

The match between the localized histograms, *h_i,j_*(*f, t*), computed from the selected and shifted image patch (bottom-up), and the analogous localized histograms, ${ĥ}_{\text{td}}\left(f,t\right)$, computed from the learned images (top-down) is determined as

(29) $${\text{match}}_{\text{sh}}\left({\text{c}}_{i,j},t\right)=\int {ĥ}_{i,j}\left(f^{\prime },t\right){ĥ}_{\text{td}}\left(f^{\prime },t\right)\;{df}^{\prime }.$$

where the “hats” again indicate mean-freeing and normalization as described by equation (4). This match is used to update the pose representation by contributing input to the dynamic neural fields representing object position (equation (9)). This requires lifting the subsampled grid-representation back to the full image sampling by bicubic interpolation.

For the color feature channel, the top-down histogram, ${ĥ}_{\text{td}}\left(f,t\right)$, is the weighted sum of the color histograms extracted from training images. For the edge orientation feature channel, the learned histograms of edge orientations extracted from the training images must first be rotated based on the current orientation estimate as described next. The weighted superposition of these rotated, learned histograms serves as top-down histogram, ${ĥ}_{\text{td}}^{\text{rot}}$.

#### 2.3.3 Rotation

In the bottom-up path, edge histograms are transformed based on the representation of object orientation, *p*_rot_(Δϕ, *t*):

(30) $$h_{\text{bu}}^{\text{rot}}\left(\phi ,t\right)=\int_{0}^{\pi }\;p_{\text{ rot}}\left(\phi -\phi^{\prime },t\right)\;h_{\text{bu}}^{\text{sh}}\left(\phi^{\prime },t\right)\;{d\phi }^{\prime }$$

where the edge orientation, ϕ ∈ [0, π), covers only half of orientation space because the edge feature does not include polarity. The top-down histogram, *h*_td_, is rotated analogously, by applying the inverse of the representation of orientation, $p_{\text{rot}}^{\text{inv}}\left(\phi -\phi^{\prime },t\right)=p_{\text{rot}}\left(\phi^{\prime }-\phi ,t\right)$:

(31) $$h_{\text{td}}^{\text{rot}}\left(\phi ,t\right)=\int_{0}^{\pi }\;p_{\text{ rot}}^{\text{inv}}\left(\phi -\phi^{\prime },t\right)h_{\text{td}}\left(\phi^{\prime },t\right)\;{d\phi }^{\prime }$$

The edge channel contributes to the update of the representation of object orientation because it is sensitive to orientation (the color channel is not and, therefore, does not contribute). The update is based on the correlation of the shifted bottom-up histogram for the edge channel, *h*^sh^, with the corresponding, non-rotated top-down histogram, *h*_td_:

(32) $${\text{match}}_{\text{rot}}\left(\phi ,t\right)=\int_{0}^{\pi }\;h_{\text{bu}}^{\text{sh}}\left(\phi +\phi^{\prime },t\right)h_{\text{td}}\left(\phi^{\prime },t\right)\;{d\phi }^{\prime }.$$

This match function contributes input to the dynamic neural fields representing object orientation (equation (9)).

#### 2.3.4 Matching Histograms to Object Views

In order to estimate object identity, transformed bottom-up histograms, *h*_bu_, are matched against learned patterns, *W_l_*. In analogy to matching in the shape channel (see equation (3)), the histograms are matched by correlating their mean-free normalized versions:

(33) $${\text{match}}_{l}\left(t\right)=\int {ĥ}_{\text{bu}}\left(f,t\right){Ŵ}_{l}\left(f,t\right)\;\mathrm{df}.$$

For the color channel, *f* = *c* is a color and $h_{\text{bu}}\left(c,t\right)=h_{\text{bu}}^{\text{sh}}\left(c,t\right)$. For the edge orientation channels, *f* = ϕ is an edge orientation and $h_{\text{bu}}\left(\phi ,t\right)=h_{\text{bu}}^{\text{rot}}\left(\phi ,t\right)$. The match function contributes input to the dynamic neural nodes representing object identity (equation (17)).

### 2.4 Fusing the Different Feature Channels

Evidence for object identity and pose from the five feature channels is fused by weighted addition. Each channel contributes to the input, *s*_1_(*r, t*), of the dynamic neural fields that represent the different dimensions, *r*, and analogously to the input of the label nodes. Different channels contribute to different dimensions of pose, as illustrated in Figure 3. The color channel is invariant under rotation and thus only contributes to the estimation of position. The localized histograms of edge orientations are extracted on three different color channels and contribute to the estimation of position and orientation. The shape-based channel also contributes to these two dimensions. In principle, shape may provide an estimate orientation across the complete range of orientation from 0° to 360°. Because the contribution of shape to orientation is relatively weak, we did not use it to disambiguate the orientation estimate delivered by the edge channel, which cannot distinguish between an image and its rotation by 180°. Instead, we restrict the orientation estimate across all channels to the range of 0° to 180°. For shape, this means that we sum the activations in the two sub-intervals of that orientation space. The discriminative power of the shape channel is also relatively weak so that its contribution to object identification is less important than its contribution to pose estimation.

### 2.5 Learning Objects

The architecture learns objects during a supervised training phase. In this phase, object images are presented to the system one by one. For each image, the system’s continuous-time dynamics is simulated in the same way as during recognition. Because at that time, the object has not yet been learned, the question arises how its pose is being represented. We set the pose in all transformation fields by biasing the first-layer pose representation fields with Gaussian inputs centered on zero. These inputs are strong enough to induce a peak at zero, which then drives the second layer as well. In that layer, pose values that become activated by the matching processes lose the competition mediated by global inhibition. As a result, the pose of the object to be learned is defined as zero. The mean shift and rotation applied to the input pattern is zero, although the width of the Gaussians induces a slight blurring to the transformed patterns.

Analogously, object identity is set by biasing a dynamic neural node on the first layer of the object identity representation (see Section 2.2.1). This reflects an externally cued label, *l*_cued_. The cued node becomes active and drives the corresponding node on the second layer, where all other nodes are suppressed by global inhibition.

For each feature channel and each label, *l*, a memory of a feature pattern, *W_l_*(*f, t*), is learned on a timescale, τ_learn_, by the learning dynamics:

(34) $$\tau_{\text{learn}}{Ẇ}_{l}\left(f,t\right)=-\left(W_{l}\left(f,t\right)-m\left(f,t\right)\right)b_{\text{learn}}\left(t\right)p_{l}\left(t\right).$$

The linear term, −(*W_l_*(*f, t*) −*m*(*f, t*)), creates an attractor for the learned feature pattern, *W_l_*(*f, t*), at the feature pattern *m*(*f, t*) that results from the bottom-up path summarized below. The factor, *b*_learn_(*t*) ∈ {0, 1}, enables and disables learning and is controlled externally, and initially set to zero. Once the pose and label estimates converge, *b*_learn_(*t*) is set to one to allow the learned pattern to converge to *m*(*f, t*) over time. *p_l_*(*t*) is the current object identity representation defined in equation (21). After the object identity representation has converged, the cued label, *l*_cued_, is represented by $p_{l_{\text{cued}}}$(*t*) = 1, with $p_{l\prime }\left(t\right)$ = 0 for all *l*′ ≠ *l*_cued_. As an effect, only the learned pattern for the cued label changes.

The feature pattern to be learned, *m*(*f, t*), is always the fully transformed pattern of the bottom-up path of the feature channel. For the color histogram, *f* = *c* is a color and $m(c,t)=h_{\text{bu}}^{\text{sh}}\left(c,t\right)$. For the edge orientation channels, *f* = ϕ is an edge orientation and $m\left(\phi ,t\right)=h_{\text{bu}}^{\text{rot}}\left(\phi ,t\right)$. For the shape channel, *f* = (*x, y*) is a spatial location and *m*(*x, y,t*) = *I*^rot^(*x, y, t*).

### 2.6 Evaluation Methods

Before evaluation, the architecture is trained in a number of training trials. In each training trial, a single training image is presented to the architecture. At the start of each trial, the architecture undergoes a soft reset in which the resting level of all fields and nodes is lowered, leading to a decay of their activation. Once their activation has fully decayed so that the activity of all field sites and nodes is sufficiently close to the resting level, the reset is considered complete, and the architecture converges, reflecting the specified pose and label information as described in Section 2.5. Once the label representation has converged, the learned patterns are adapted as described in Section 2.5. This learning phase has a fixed duration after which the training trial is considered complete, and the next trial starts.

Once the architecture is fully trained, recognition performance is assessed. Recognition trials begin with the same soft reset procedure as training trials, after which a query image is presented and the system is allowed to converge to a pose and label estimate. The recognition process is considered complete when the activation of a label node on the second layer remains above threshold for a fixed time interval or once trial duration exceeds a given maximum. The estimated pose and identity are recorded, and the next image is processed.

We explain next how recognition performance is assessed through a rank measure and then describe how pose estimation is assessed. Finally, we describe a simplified recognition model without pose estimation that is used to assess how the different components contribute to the performance of the model.

#### 2.6.1 Rank Measure

At the end of each recognition trial, we record the output of the second layer of label nodes, σ(*u*_2,*l*_(*t*)), where *l* is a label. We rank order labels by their output level into a list (*l*_1_, *l*_2_, …, *l_n_*) with *l_i_* ≠ *l_j_* and $\sigma \left(u_{2,l_{i}}\left(t\right)\right)\geq \sigma \left(u_{2,l_{i+1}}\left(t\right)\right)$ ∀*i* ∈ {1, …, *n* − 1}, so that:

(35) $$\text{rank}\left(l_{i}\right)=i.$$

The label, *l*, for which rank (*l*) = 1, is the best-matching label; the label, *l*, for which rank (*l*) = 2 is the second-best match; and so on. When the best-matching label corresponds to the annotated label of the presented view, the trial counts as a correct recognition.

#### 2.6.2 Measuring Pose Errors

At the end of each recognition trial (indicated by the time, *t*_end_), we also record the output of the second layer of the pose estimation fields, *u*_2_(*r, t*) (where *r* is a pose dimension), which manifest a localized peak of activation at this time. The location of these peaks provide the pose estimates. For position, the pose estimate

(36) $$\tilde{\text{x}}=\left(\tilde{x},\tilde{y}\right)=\underset{x,y}{\text{arg}\;\text{max}}\;\sigma \left(u_{2}\left(x,y,t_{\text{end}}\right)\right)$$

leads to a pose error computed as

(37) $$E_{\text{sh}}\left(\text{x},\tilde{\text{x}}\right)=\sqrt{{\left(x-\tilde{x}\right)}^{2}+{\left(y-\tilde{y}\right)}^{2}},$$

where **x** = (*x, y*) is the annotated position of the object. For orientation, the pose estimate

(38) $$\tilde{\phi }=\underset{\tilde{\phi }}{\text{arg}\;\text{max}}\;\sigma \left(u_{2}\left(\tilde{\phi },t_{\text{end}}\right)\right).$$

leads to a pose error of

(39) $$\begin{array}{l} E_{\text{rot}}\left(\phi ,\tilde{\phi }\right)=\left\{\begin{array}{ll} |\tilde{\phi }-\phi |\; & |\tilde{\phi }-\phi |<90^{{}^{\circ}}\\ 180-|\tilde{\phi }-\phi |\; & \text{otherwise} \end{array}\right., \end{array}$$

where ϕ ∈ [0°, 180°] is the annotated orientation of the object.

#### 2.6.3 Role of Pose Estimation in Recognition

One question is how much the concurrent estimation of pose and of object identity contributes to recognition as compared to the raw recognition rates that can be obtained from the feature channels alone, without concurrent pose estimation. To address this question, we implemented a variant of the recognition approach, in which a nearest neighbor classification scheme employs the same color and edge channels as used in our main model. In this scheme, histograms, *h_F,i_*, for each feature channel, F, are extracted from each training image indexed by *i* according to

(40) $$h_{F,i}\left(f\right)=\iint \delta \left(f-I_{F,i}\left(x,y\right)\right)\;\mathrm{dx}\;\mathrm{dy}.$$

where *I_F,i_*(*x, y*) is the spatial pattern of feature values in the training image. (Note that this corresponds to equation (22) without the local Gaussian and the top-down weights.) The correlation between the query image and each training image (index, *i*), $\int {ĥ}_{F,\text{query}}\left(f\right){ĥ}_{F,i}\left(f\right)\mathrm{df}$ (the “hat” indicates mean-freeing and normalizing of each histogram) is combined across feature channels, *F*, with weights, *w_F_*. The best-matching training image,

(41) $$\begin{array}{l} i^{*}=\underset{i}{\text{arg}\;\text{max}}\;\sum_{F}\;w_{F}\int {ĥ}_{F,\text{query}}\left(f\right){ĥ}_{F,i}\left(f\right)\;\mathrm{df} \end{array}$$

is recognized. We tested two ways to combine feature channels. The *no-pose-color* approach (NP-C) uses only color information (i.e., *w*_col_ = 1, all other weights zero). The *no-pose-color and edges* (NP-C + E) comparison uses color information as well as edge orientations on the black (Y) and chroma components (Cb and Cr) of the input image. The contributions of these feature channels, *w_F_*, are given the same weight as in the proposed system.

We first tested the new approach on the COIL-100 dataset (Nene et al., 1996), a well established benchmark for object recognition. Because objects in COIL are rotated outside the image plane, the assumptions on which pose estimation is based in the proposed system are violated. We provide, therefore, a new dataset on which we are able to make detailed quantitative assessments of both object identity and pose estimation. All evaluations presented here are based on an implementation of the proposed system in the software framework *cedar* (Lomp et al., 2013).

#### 2.6.4 Baseline Comparision

To provide a baseline for the recognition performance of our model, we employed the SURF-based object recognition system (Bay et al., 2008). This well-established approach achieves approximate pose invariance through rotation and scale invariant interest point descriptors. Rotation and orientation estimates can be obtained from the system. We applied the method to both out tabletop images and to the COIL data set.

## 3 Results

### 3.1 Evaluation on the COIL-100 Dataset

The COIL-100 database (Nene et al., 1996) consists of color images of 100 objects, each recorded individually in front of a uniform, dark background from one of 72 different view angles achieved by placing the object on a turntable that was rotated in 5° intervals. Our model recognizes objects based on a single view. To approach the COIL paradigm, we used four training views for each object, taken from 0°, 90°, 180°, and 270° angles. Each training view is represented by a dynamic neural node. The four nodes representing the four views of a single object are combined in a second two-layer stage of neural dynamics at which one node stands for one object. We only learned the first 30 objects of COIL-100.

We added a uniform border of 64 pixels to the images. The color of this border matched the background color of the COIL-100 images to avoid artifacts from edge responses at the border of the padded image. Padding the images allowed us to keep sampling rates and other parameter values in our architecture the same as in the experiments performed in the next section, in which we will locate objects in 256 × 256 pixel images with a relatively large amount of background.

The set of query images was presented in different random orders four times, while the recognition process ran in one continuous simulation. In this scenario, the proposed system achieved a recognition rate of 91.1%. The recognition rate achieved by the SURF baseline method on the same set of images was 57.8%.

The impact of pose estimation on the recognition rate is summarized in Table 1. Using color histograms alone, without pose estimation, (NP-C) leads to a higher recognition rate than that of our proposed method. When edges are added to color (NP-C + E), the advantage goes away and the recognition rate approximately matches that of our proposed method. These two observations may at first seem puzzling. That color performs well reflects the invariance properties of color histograms. Edges are less discriminative and less invariant, so adding the edge feature degrades performance. Color histograms do not enable orientation estimation, of course, so our proposed method needed to include the less discriminative edge feature. However, on the COIL data base, estimating the pose does not improve performance. This is probably due to the fact that most pose variation in the COIL database comes from rotations in depth, which are not estimated in our approach. In the tabletop setting, in contrast, the variation of pose is better captured by the image-based pose estimation process of our approach.

**Table 1:** **Overview of the recognition performance on the test portion of the datasets**.

Database	COIL (first 30) (%)	Tabletop (%)
Proposed system	91.1	87.2
SURF baseline	57.8	34.8
NP-C	95.3	84.4
NP-C + E	92.2	81.5

*Please refer to Section 2.6.3 for an explanation of the “no-pose” (NP) variants. Note that in order to capture stochastic variations in the recognition results that stem from the noise in the dynamics of the pose and label representations, these results are averaged over four runs through the set of test images*.

### 3.2 Tabletop Setting

**Figure 6:**
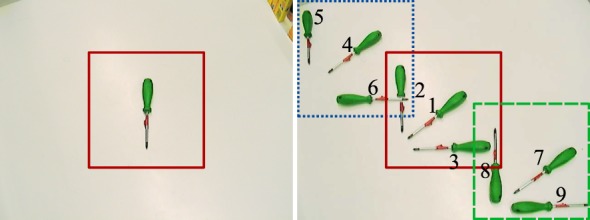
**The ten poses used in the tabletop scenario, here shown for the *green screwdriver***. Left: in the training pose, the object’s long axis is aligned with the vertical image axis. The solid red square indicates the 256 × 256 subregion of the image that is used for training. Right: all different poses superimposed in one image. Poses are numbered 1–9 as shown in the figure. The different squares indicate the subregions of the image used as input to the recognition process; the dotted (blue) region is used for poses 4–6, the solid (red) region is used for training and poses 1–3, and the dashed (green) region is used for poses 7–9.

A tabletop dataset that specifically probes both object identity and pose estimation was previously developed in preliminary work (Faubel and Schöner, 2008, 2009). It contains images of thirty common household objects in a robotic tabletop setting. Each image shows an object in one of ten different positions on a white tabletop in front of the robotic platform CoRA (Iossifidis et al., 2003). Of the ten positions, one is the training pose, while the other nine are used for testing (see Figure 6). The images are captured by a Sony DFW-VL500 camera with a resolution of 640 × 480 pixels. Lighting conditions as well as camera position and settings are constant throughout the whole dataset.

For the recognition process, a subregion of 256 × 256 pixels is cut out from the images. The cut out region is placed in the center of the full-sized image for poses 1–3, at the top-left corner for poses 4–6, and at the bottom-right corner for poses 7–9 (see Figure 6). This cutting-out procedure is meant to reflect the effect of attention that would focus input to the recognition system on the vicinity of the object to be recognized.

**Figure 7:**
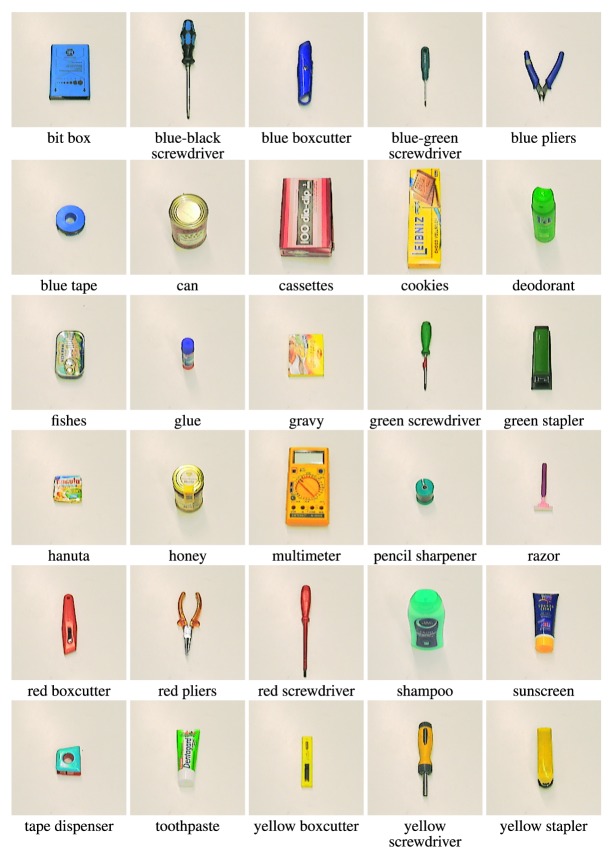
**Training views of the 30 objects of the tabletop database**.

Figure 7 shows cropped training images of all objects in the database. The full images and pose annotations of the tabletop dataset are available online (see text footnote 1).

Note that in contrast to many other databases, objects take up only a small portion of the input image due to the camera’s angle of view (see Figure 6 for examples). The problem of foreground-background segmentation is simplified by presenting objects on a white tabletop. Training views show objects in a canonical pose, that is, with their longer axis pointing up, in order to simplify the calculation of pose errors (if objects were not learned in canonical poses, we would have to record, for each object, what the estimated pose was during learning, and computing the error would need to reference this recorded estimate).

#### 3.2.1 Recognition and Pose Estimation Performance

We first trained the system using the 30 training images from the tabletop scenario. Each training image was presented a single time, so that the original form of the architecture could be used. As in the COIL experiments, results from four runs through the entire test set, in randomly permuted order, were averaged to compute recognition and pose estimation performance.

The recognition rate of our system was 87.2%. This compares to the baseline SURF system which achieved a recognition rate of 34.8%. This is a stronger contrast than for the COIL data base. The tabletop database entails more strongly the type of pose variation that our system is able to estimate.

Table 1 compares recognition performance of the system to the two variants that do not estimate pose. Note that for the table top scenario, pose estimation is always advantageous. Given that our system uses edges as features to enable the estimation of rotation, the contrast of our system to NP-C + E is a direct assessment of the advantage of pose estimation provides, approximately a boost of 6% in recognition rate. Recognition based on color alone (NP-C) is still better than recognition that also includes the less discriminative and invariant edge information (NP-C + E).

The performance of the proposed system on pose estimation is compared to the SURF baseline method in Table 2. The proposed system dramatically outperforms the baseline.

**Table 2:** **Pose estimation errors**.

	Proposed method		SURF baseline	
	All	Correct	All	Correct
Position (px)	13.5	13.0	52.4	87.3
Rotation (°)	14.0	12.1	37.1	30.5

*Errors have been averaged separately over *all* test trials as well as those test trials in which the *correct* object was recognized. Please refer to the text for details on how these errors are calculated*.

**Figure 8:**
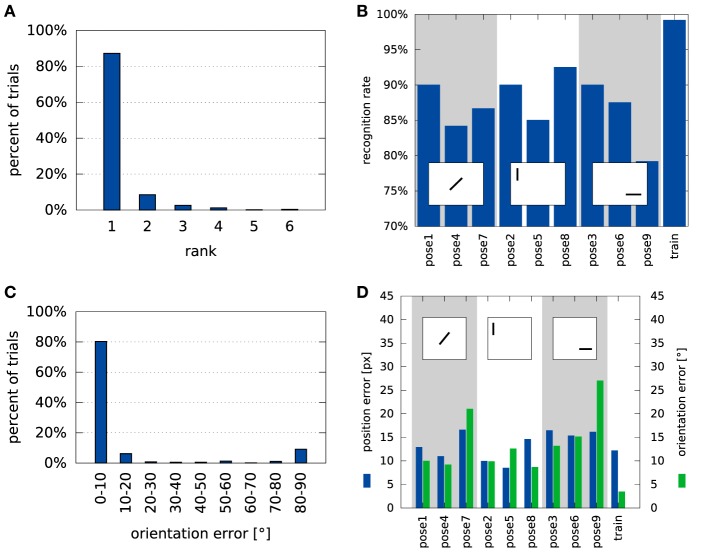
**Overview of the system’s performance on the tabletop dataset**. Panel **(A)** shows a histogram of ranks of the correct label in the test trials (see Section 2.6.1 for a description of the rank measure). Panel **(B)** shows recognition performance by pose. Please refer to Figure 6 for an explanation of the different poses and Section 2.6.2 for an explanation of how pose errors were calculated. Panel **(C)** shows a histogram of rotation errors. **(D)** Details the position errors for each of the object poses in the database. The square inlays in **(B,D)** symbolize the position and alignment of the object in the views, relative to the cutout region.

Figure 8 provides a more detailed characterization of recognition and pose estimation performance. The distribution of the rank of the correct label across all test trials shown in Figure 8A indicates that the average rank of the correct class was 1.2. Within the set of trials in which the object was incorrectly classified, the average rank of the correct class was 2.5.

Figure 8B illustrates how recognition rates varied with the deviation from the training pose. Shifted (left) and rotated (center) poses somewhat degrade performance, most strongly when shift is combined with a large rotation (right).

Figure 8C shows how the error of orientation estimation is distributed. Note, that the maximum error is 90° because rotation was only estimated within the range [0°, 180°] (see equation (39)). The small peak around 90° reflects that objects tend to have two main axes, which may be confused when objects are approximately quadratic. Finally, the dependence of these errors on the tested poses is shown in Figure 8D. It shows a similar pattern as the recognition rates, with the largest errors occurring when a shift and a large rotation are combined (right).

To assess the contribution of each feature channel to the performance of the model, we deactivated all but one feature channel (by setting all weights to zero except one, which was reweighted to achieve the same overall input strength). Table 3 lists measures of pose and recognition performance for each individual feature channel. Color histograms make by far the strongest contribution to recognition, but contribute nothing to the estimation of orientation (45° being random for a uniform distribution from 0° to 180°), both expected outcomes. However, color histograms contribute the best estimation of shift. Edges contribute to the estimation of orientation, most strongly for the luma-based edges (Y). Color edges contribute a little more to recognition than the luma-based edges. The shape channel is useful mainly for shift estimation.

**Table 3:** **Performance of the individual feature channels on the tabletop dataset**.

		Pose errors	
Channel	Recognition rate (%)	Position (px)	Orientation (°)
Color	85.2	17.0	44.9
Y edges	7.7	21.4	14.4
Cr edges	11.3	28.8	24.4
Cb edges	11.4	27.8	21.2
Shape	6.3	20.3	42.1

#### 3.2.2 Feature Sampling

**Figure 9:**
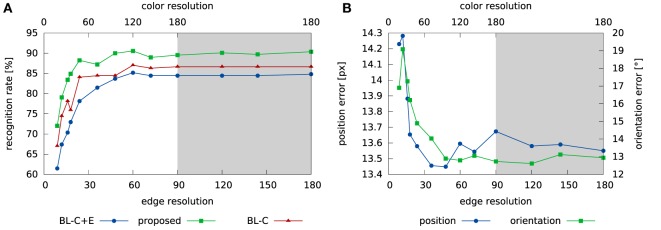
**This figure shows how the resolution of feature sampling affects the performance of the system on the tabletop images**. Panel **(A)** shows the impact on the recognition rate, while panel **(B)** shows how pose estimation performance changes. The *x*-axis indicate the number of points used to sample the continuous feature values, *f*, in equation (22). Note that the color feature channel is sampled at double the resolution of the edge feature, with a maximum resolution of 180 feature bins. The gray area in the plots marks cases where color resolution is maximal.

The performance of the system depends on the resolution of the feature histograms. In order to quantify this dependence, we used the same training- and testing procedure but varied the resolution of the color and edge feature channels. The resulting recognition performance is shown in Figure 9A, while the pose estimation errors are shown in Figure 9B.

#### 3.2.3 Partial Estimation

To separately evaluate the system’s pose and identity estimation performance, we provided either location or object identity information to the system on test. For *label only* recognition, pose information was provided by Gaussian inputs into the pose representation fields centered on the veridical pose. Pose representation fields are thus strongly biased to select the specified pose. As a result, pose estimation converges to the correct value early in the recognition process. For *pose only* recognition, the correct label node receives a strong bias. The weighted superposition of learned object views, therefore, approximates the correct top-down prediction very early in the recognition process.

**Figure 10:**
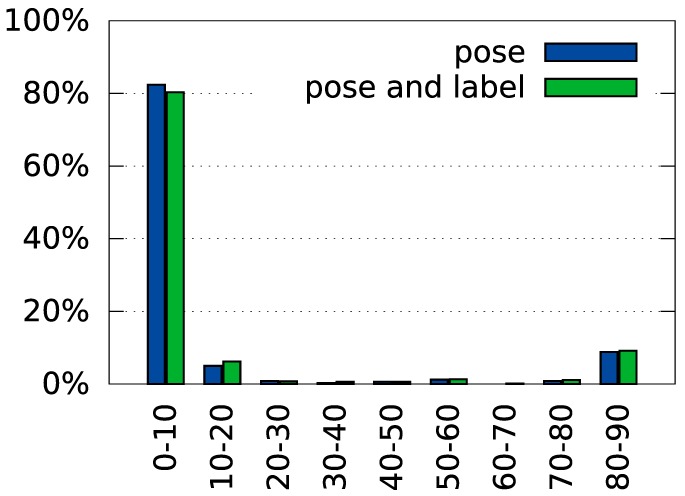
**Histogram of rotation errors with partial supervision**. In the “pose” condition, the correct label is specified during recognition. In the “pose and label” condition, no additional information is given to the system (values reproduced from Figure 8C for comparison).

**Figure 11:**
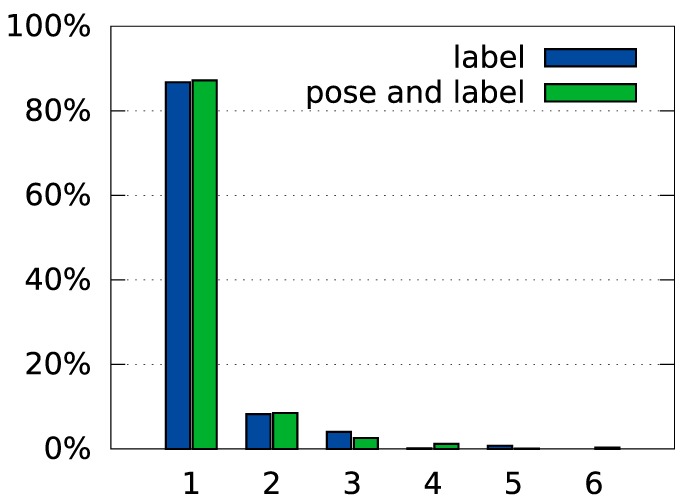
**Histogram of ranks when only labels are estimated**. In the “label” condition, the correct pose is specified during recognition. In the “pose and label” condition, no additional information is given to the system (values reproduced from Figure 8A for comparison).

Table 4 compares performance in these two variants against the combined pose and identity estimation process. Figure 10 shows a histogram of rotation errors in *pose only* recognition. Figure 11 shows a distribution of ranks for *label only* recognition.

**Table 4:** **System performance when partial information on the object in the image is provided**.

	Estimated parameters		
	Pose only	Label only	Pose and label
Recognition rate (%)	100.0	**86.8**	**87.2**
Position error (px)	**13.0**	0.8	**13.5**
Orientation error (°)	**13.3**	1.0	**14.0**

*In “pose only” recognition, label information was given to the system. In “label only” recognition, pose information was provided. “Pose and label” recognition is the concurrent estimation of both object pose and identity (results are taken from Table 1). Numbers printed in bold face correspond to information that was estimated by the system in the testing phase*.

#### 3.2.4 Multiple Objects in One Image

**Figure 12:**
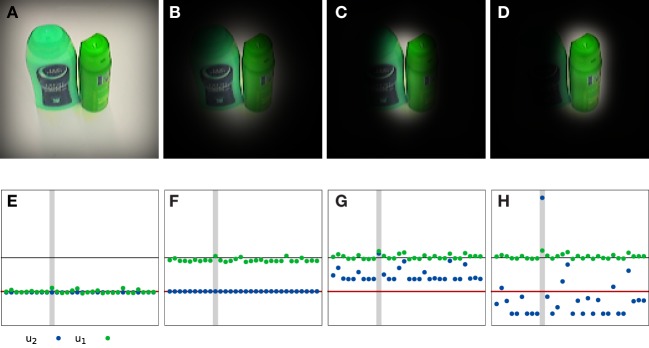
**An example of a recognition trial with an input image that contains multiple known objects**. Top row: result of masking the input image with the currently estimated top-down shape at different times of the recognition process [*t* = 0 s **(A)**, *t* = 1 s **(B)**, *t* = 2 s **(C)**, and *t* = 7 s **(D)**]. Bottom row: corresponding activation of the nodes on the first (green) and second (blue) label layer [*t* = 0 s **(E)**, *t* = 1 s **(F)**, *t* = 2 s **(G)**, and *t* = 7 s **(H)**]. The red line marks the activation level to which the nodes converge during the reset. The black line marks the threshold for considering a node active. The gray bar indicates the node corresponding to the label on which the system settles in the end. Two videos of this process are available in the Supplementary Material, one showing the process at full speed and one showing it at a slower speed.

Figure 12 demonstrates that the proposed system is capable of focusing on a single object even if the test image contains multiple objects. This is ultimately the result of the top-down path that effectively provides a variable mask on top-down input. To see this, Figure 12 provides a timeline of the recognition process in such a situation. At the end of the soft reset of the system (in Figure 12A around *t* = 0), all pose representation fields and object nodes are below threshold and the predicted top-down shape is broad and spans the whole image. After a short time, the position and orientation estimates begin to sharpen. This filters out some of the irrelevant information and thus helps refine the label estimate. The shape estimate becomes more localized (Figure 12B). However, there are still multiple candidates for the object being recognized (Figure 12F), and the top-down shape contains contributions from all of these candidates. Shortly afterward, the system decides for a label (Figure 12G). All other candidates are suppressed, and as a result, the top-down shape begins to reflect the selected object only (Figure 12C). This decision enables the system to further refine the pose estimates until they converge to the pose of the recognized object (Figure 12D).

#### 3.2.5 Tracking

**Figure 13:**
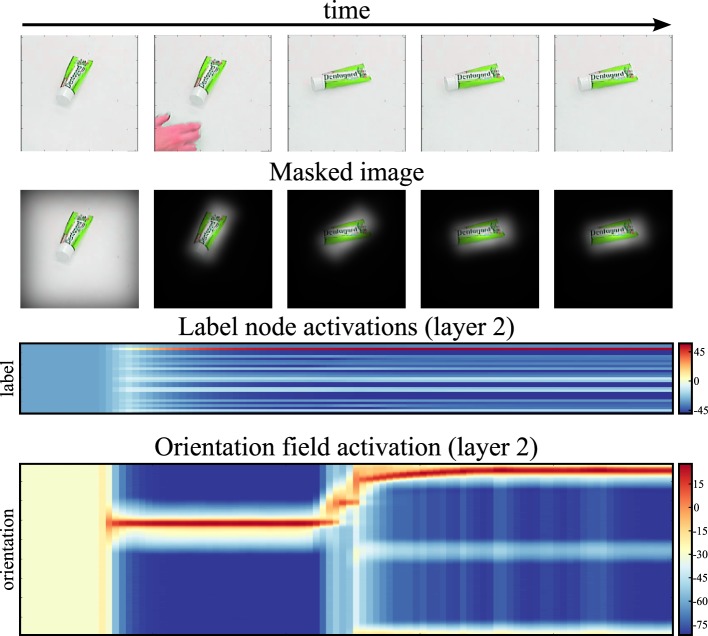
**This figure shows our system tracking an object in a video in real time**. The first row of images shows the input stream at select time steps. The second row of images shows the input region, masked by the top-down shape estimate. The third row shows the activation of the label nodes over time, and the fourth row shows the time-course of the orientation estimate.

Figure 13 shows the system tracking a moving object in real time. Initially, the object is placed at the center of the image. After the system has recognized the object, the user rotates it. Note that the recognition decision persists (label activation stays above threshold), even though the user’s hand touching the object changes the image within the viewing area that provides input to the recognition system. Persistence of object identity estimation removes the need to restart the recognition process for each frame. Instead, the recognition system smoothly tracks the changes in the object’s orientation. This scenario highlights an important property of the neural dynamics on which this approach is based: recognition decisions are stabilized over time. Masking the input with the current shape estimate further stabilizes the recognition decision by suppressing visual input outside the object boundaries.

## 4 Discussion and Conclusion

In this paper, we have developed a system that learns to identify objects from single training views. In a typical tabletop scenario, the system will identify these objects as they are handled by a human or a robot, while at the same time estimating the objects’ current pose relative to the pose in the training view. The system is based on ideas from neural dynamics, in which a recurrent loop of top-down and bottom-up processing generates stable states for both pose estimation and object identification.

We have evaluated the recognition performance of the system in a tabletop scenario and found correct identification in 87.2% of all trials, much better than the SURF baseline which clocked in at 34.8%. Even when the object is not correctly identified, the correct object label is among the top choices of the system (see Figure 8A). Although much of the discriminative power of the system comes from the features used, concurrent pose estimation increases this power in the tabletop scenario, especially when features are sampled coarsely (Figure 9A). In the COIL database, variation of pose is not captured by shift and image-based rotation as strongly. As a result, pose estimation did not improve recognition performance. The recognition rate was still respectable (91.1%), better than the SURF base line (57.84%), but lower than with color histograms alone.

Evaluating the performance in pose estimation for the tabletop database, we found that the pose estimation error was small on average (Table 2). For object orientation, most trials led to an estimation error of less than 10°. Errors around 90° deviation (Figure 8C) occur with some frequency, reflecting the symmetry properties of some objects (e.g., square objects that have the same edge distribution when rotated by 90° or round objects that do not have a clear long-axis on which orientation can be anchored). Discarding these special cases, the precision of pose estimation becomes impressive, given that pose estimation is view based and thus approximate only.

The need to estimate object pose does not generally limit recognition performance. When full pose information is given, recognition performance is not improved (see Table 4). This suggests that the annotated poses are not more accurate than the estimates delivered by the system.

The processing speed in our implementation of the architecture is sufficient to deal with real time camera input. This speed could be improved by optimizing the computational implementations. In particular, the computationally expensive construction of the localized histograms could be parallelized to further improve real time performance.

There are neurally inspired object recognition approaches that may achieve higher recognition rates. For example, deep networks reach very high recognition rates that may exceed human performance (Ciresan et al., 2012). These approaches cannot be meaningfully compared to the system studied here, however, because they address a different task. Typically, these approaches recognize objects in the sense of assigning a new instance to a learned object class that has been extensively trained using a variety of training examples. In contrast, the system we developed here learns object from a single view stored in a single shot learning procedure. When the system recognizes an object, it not only estimates object identity but also object pose. Most neurally inspired approaches do not address pose estimation. When object recognition is combined with pose estimation in scenarios similar to ours, recognition rates are low (e.g., below 70% for 28 objects (Kragic et al., 2005)).

A successful branch of feedforward neural networks based on the HMAX model (Riesenhuber and Poggio, 1999; Serre et al., 2007) achieve pose invariant object recognition by making features invariant against changes of pose in a multilayer architecture. The system developed here achieves pose invariant recognition, instead, by explicitly estimating pose, so that the transformation of a current view to the learned view becomes possible. This requires features that support such active pose transformation. For example, localized color histograms vary when objects are shifted, but not when they are rotated because their receptive fields are point symmetric around their center. Localized color histograms are thus used to estimate object position, but not object orientation. Localized histograms of edge orientation, in contrast, vary both when objects are shifted and rotated and are thus used to estimate both object position and orientation. Conversely, top-down prediction of views from learned views requires a neural representation of object identity from which views can be constructed by weighted superposition.

SIFT based approaches come closest to what we have reported here. In application to robotic scenarios, they typically use 3D estimation (e.g., from multiple cameras as in Collet et al. (2011), or RGB-D cameras as in Schoeler et al. (2014)) and focus less on single view-based recognition. In preliminary work, we have used keypoints within our framework and found that at tolerable computational effort the recognition rate was lower (Lomp et al., 2014).

The concurrent estimation of pose and object identity endows the system with additional functionality. For instance, segmentation of the visual array into the foreground object and background distractors emerges from the system’s dynamics. This is illustrated in Figure 12, which tracks the time course of recognition and pose estimation when two objects are in the field of view. As the system converges toward correct identification and pose estimation, the mask that the top-down path applies to the input image focuses on the identified object and suppresses the distractor object. This figure-ground segmentation not only stabilizes the recognition decision but also prevents interference from other parts of the visual array, such as from the human or robotic hand that may be visible while handling the object.

Such resistance to distractors is demonstrated in Figure 13 along with the system’s capability to track changes in the input image. In the demonstration, after initial recognition of the object, a human operator rotates the object by hand. Due to the masking and stabilizing properties of the neural dynamics, the visible hand has no bearing on the recognized label. The changing object orientation is smoothly tracked as the neural dynamics keeps converging to the moving attractor. Tracking is an emergent property of the dynamics of the neural field, in which local excitatory interaction actively supports the update of the location of activation peaks as inputs shift. Online tracking of object poses is critical in scenarios in which objects are handled such as in the toaster repair scenario we alluded to in the Introduction. A simplified version of the developed system has, in fact, been deployed in a similar scenario (Knips et al., 2014).

More generally, the system developed in this paper is an exemplary integration of feed-forward neural architectures with a recurrent loop of top-down prediction. The feed-forward or bottom-up path, provides high-dimensional feature information, here color and edge orientation distributions, that endows the model with discriminative power. The recurrent processes of estimating pose and object identity are enabled by neural dynamics that provide the competitive interaction necessary to filter out non-matching pose and object identity candidates over time and stabilize the resulting selection decision. Among possible extensions of the approach is the introduction of an additional level of transformation for scale, which amounts to a shift transformation along the distance dimension of the log-polar representation of the current model.

We have seen that feature channels are complementary. Edge features are good at pose estimation but not very discriminative, while color is most discriminative for recognition but provides no orientation information. Combining the different feature channels is thus attractive. The neural-dynamic framework is particularly well suited to achieve this combination in closed loop. Tracking exploits the stability of pose estimation in neural dynamics and leverages the strength of the feature channels most suited to pose estimation.

## Author Contributions

GS and CF designed the research plan. CF developed a first implementation and evaluated it. OL developed the final implementation and performed the experiments reported here. All three authors contributed to writing the paper.

## Conflict of Interest Statement

The authors declare that the research was conducted in the absence of any commercial or financial relationships that could be construed as a potential conflict of interest.

## Footnotes

^1^https://www.ini.rub.de/pages/publications/LompFaubelSchoener2016.

## Supplementary Material

Video S1 **The video shows the system recognizing an object when two known objects are shown in the input image at normal speed**.

Video S2 **The video shows the system recognizing an object when two known objects are shown in the input image**. The process is slowed down to 5% of real time.
