# Supplementary material for: A Neural-Dynamic Architecture for Concurrent Estimation of Object Pose and Identity
Source: Front Neurorobot. 2017 Apr 28;11:23. doi: 10.3389/fnbot.2017.00023 (PMC5408094; doi:10.3389/fnbot.2017.00023)
Supplement: Supplementary file 3 [file Data_Sheet_1.PDF]

Supplementary Material: A neural-dynamic architecture for concurrent estimation of object pose and identity

Oliver Lomp *, Christian Faubel, and Gregor Schöner

*Correspondence:
Oliver Lomp
oliver.lomp@ini.ruhr-uni-bochum.de

S1 PARAMETER VALUES

In the present section, we detail the chosen values for the parameters of our model that are left out in the main paper to avoid clutter. Parameters are presented roughly in the order of mention in the main text.

S1.1 Field parameters

**field**	τ_i [ms]	h_i	a_1	σ_1	a_2	σ_2	γ_i	β	c_{η_i}
shift layer 1	30	-1.7	15.0	10.0	-14.9	40.0	-0.003	11.0	0.1
shift layer 2	100	-1.0	13.0	2.0	-49.0	15.0	-0.3	100.0	1.5
rotation layer 1	50	0.0	2.0	5.0	-0.8	20.0	-0.01	20.0	0.01
rotation layer 2	300	-1.0	60.0	2.01	-80.0	8.0	-11.85	1000.0	1.5

**connection**	c_{12}	σ_{12}
shift layer 1 to layer 2	2.75	1.0
rotation layer 1 to layer 2	8.0	1.0

S1.2 Label node parameters

**node layer**	τ_i [ms]	h_i	$w_{i,\text{self}}$	γ_i	β	$c^{\text{L}}_{\eta,i}$
label layer 1	100	0.0	1.75	-0.001	10	0.1
label layer 2	1500	-0.7	50.0	-50.0	800	1.5

Connection strength from layer 1 to layer 2: $c_{12} = 9.0$

S1.3 Peak detectors

**peak detector**	τ_p [ms]	h_p	w_p	β
labels	150	-0.5	1.0	100.0
shift	75	-20.0	2.5	100
rotation	115	-0.5	1.0	115

S1.4 Histogram extraction

Number of grid points, $\mathbf{c}_{i,j}$, for histogram extraction: $9 \times 9 = 81$.

**channel**	σ_h
color	30.0
Y edges	30.0
Cr edges	30.0
Cb edges	30.0

**input**	**threshold**
saturation (θ_{sat})	37
value (θ_{val})	24
edge energy (θ_{edge})	100

S1.5 Channel contributions

**channel**	**label**	**shift**	**rotation**
color	7.0	6.0	—
Y edges	1.0	0.35	35.0
Cr edges	1.0	0.35	4.0
Cb edges	1.0	0.35	4.0
shape	9.0	20.0	20.0

S1.6 View memory

The learning timescale, τ_{learn}, is set to 1.25.
